# Advancing progress on tobacco control in low-income and middle-income countries through economic analysis

**DOI:** 10.1136/tc-2023-058335

**Published:** 2024-05-02

**Authors:** Roy Small, Rachel Nugent, Douglas Webb, Brian Hutchinson, Garrison Spencer, Carrie Ngongo, Roman Chestnov, Dudley Tarlton

**Affiliations:** 1 HIV, Health and Development Group, United Nations Development Programme, New York, New York, USA; 2 Department of Global Health, University of Washington, Seattle, Washington, USA; 3 RTI International, Research Triangle Park, North Carolina, USA; 4 United Nations Development Programme, Tbilsi, Georgia; 5 Independent Consultant, Geneva, Switzerland; 6 United Nations Development Programme, Istanbul, Turkey

**Keywords:** Economics, Low/Middle income country, Advocacy

## Abstract

**Background:**

More than 80% of the world’s 1.3 billion tobacco users live in low-income and middle-income countries (LMICs), where progress to address tobacco and its harms has been slow. The perception that tobacco control detracts from economic priorities has impeded progress. The Secretariat of the WHO Framework Convention on Tobacco Control (FCTC) is leading the FCTC 2030 project, which includes technical assistance to LMICs to analyse the economic costs of tobacco use and the benefits of tobacco control.

**Methods:**

The Secretariat of the WHO FCTC, United Nations Development Programme and WHO supported 21 LMICs between 2017 and 2022 to complete national investment cases to guide country implementation of the WHO FCTC, with analytical support provided by RTI International. These country-level cases combine customised estimates of tobacco’s economic impact with qualitative analysis of socio-political factors influencing tobacco control. This paper overviews the approach, observed tobacco control advancements and learnings from 21 countries: Armenia, Cabo Verde, Cambodia, Chad, Colombia, Costa Rica, El Salvador, Eswatini, Georgia, Ghana, Jordan, Laos, Madagascar, Myanmar, Nepal, Samoa, Sierra Leone, Sri Lanka, Suriname, Tunisia and Zambia.

**Results:**

Tobacco control advancements in line with investment case findings and recommendations have been observed in 17 of the 21 countries, and many have improved collaboration and policy coherence between health and economic stakeholders.

**Conclusions:**

Tobacco control must be seen as more than a health concern. Tobacco control leads to economic benefits and contributes to sustainable development. National investment cases can support country ownership and leadership to advance tobacco control.

**WHAT IS ALREADY KNOWN ON THIS TOPIC**

- Tobacco use results in significant socioeconomic harm.

**WHAT THIS STUDY ADDS**

- This paper overviews the approach, observed tobacco control advancements and learnings from 21 countries supported to complete national tobacco control investment cases.

**HOW THIS STUDY MIGHT AFFECT RESEARCH, PRACTICE OR POLICY**

- Findings can inform future directions for understanding and addressing the economic dimensions of tobacco and health.

## Introduction

Globally, in 2019, tobacco use killed nearly 9 million people and accounted for the loss of around 230 million disability-adjusted life-years.^1^ Tobacco use is a risk factor for 38 health threats, ranging from non-communicable diseases (NCDs) such as cancers and heart disease to infectious diseases such as tuberculosis and COVID-19.^2^ Tobacco’s harms afflict those who directly consume and produce it as well as people exposed to secondhand tobacco smoke, which includes nearly half of the world’s children.^3^ Tobacco’s biggest toll is in low-income and middle-income countries (LMICs). This is where 80% of the world’s 1.3 billion tobacco users live.^4 5^ Progress to address tobacco in LMICs has been slow.^4 5^

Inclusion of the WHO Framework Convention on Tobacco Control (FCTC) in the United Nations (UN) Sustainable Development Goals (SDGs) provided a breakthrough opportunity to strengthen commitment, financing and action for tobacco control. However, it also presented concerns that tobacco control could get lost as just one target amid 168 others within a broad agenda, all ostensibly competing for limited resources. This concern was justified given that reviews of sustainable development planning in multiple countries have confirmed relatively low attention to tobacco control.^6^ National investment cases for health can support countries to reframe health as an investment rather than a cost. Investment cases typically quantify the direct and indirect benefits of implementing interventions, in health and monetary terms, and weigh this against the costs of implementing the interventions. By demonstrating overall economic gains from allocating more resources to the most cost-effective interventions, investment cases have justified investments in a growing number of global health needs, from HIV/AIDS to NCDs.^7^

The FCTC 2030 project, initiated by the WHO FCTC Secretariat in 2016 and supported financially by the governments of Australia, Norway and the United Kingdom of Great Britain and Northern Ireland, supports LMICs in accelerating tobacco control to achieve the SDGs.^8^ The project highlights, measures and supports integrated action to address the multidimensional links between tobacco and sustainable development. A focus is on supporting countries to strengthen policy coherence between SDG 3 (health) and SDG 8 (economic growth). This involves challenging the false notion that tobacco is a net plus for the economy, despite its substantial health harms. This notion is perpetuated by the tobacco industry and often embraced by decision-makers and society, particularly when coupled with insufficient evidence on the economic advantages of good health.^9^

The economic case against tobacco gathered steam in the late 1990s with an influential World Bank monograph that challenged economic myths around tobacco control and continued in the early 2000s with the adoption and coming into force of the WHO FCTC.^10 11^ Reports that have estimated the global economic impacts of tobacco include the World Bank’s Economics of Tobacco (1999) and WHO’s Saving Lives, Spending Less: A Strategic Response to Non-Communicable Diseases (2018).^10 12^ Goodchild *et al* estimated that smoking-attributable diseases cost the world US$422 billion in healthcare costs in 2012 (or 5.7% of global health expenditure) and around US$1.4 trillion when adding productivity losses, equaling 1.8% of the world’s gross domestic product.^13^ (This estimate relates only to smoking, meaning that costs would be even higher if all forms of tobacco use were taken into account).^13^

Global analyses are crucial for global advocacy and informing further economic analysis. Yet they struggle to motivate national policy responses because they typically give little consideration to specific contexts and thus fail to generate buy-in from national authorities. The FCTC 2030 project fills this gap with country-led and owned investment cases. Using local data where possible, the investment cases estimate the health and economic burden of tobacco use (in the tradition of cost-of-illness studies) and evaluate the return on investment of tobacco control demand reduction measures—assessing both the state of the problem and a pathway forward. The cases also include qualitative analysis of the political economy surrounding tobacco control in each country and describe options for health ministries to navigate the policy process, lead stakeholder engagement and champion action across sectors. Often, the tobacco control investment cases offered through the FCTC 2030 project present the first-ever national picture of the economics of tobacco.

This collection of papers describes 21 national tobacco control investment cases completed between 2017 and 2022 as part of the FCTC 2030 project. This paper describes the approach and recently observed advancements on tobacco control in these countries that align with investment case findings and recommendations. It also explores learnings based on the authors’ experiences in conducting the cases to inform future directions for investment cases for health. The subsequent papers in the collection describe the methods, results and equity dimensions from the national tobacco control investment cases. Specifically, Nugent *et al* describe the economic modelling methods used, including how they are informed by a long tradition of earlier tobacco control economics work while building in the flexibility for use in varied countries.^14^ Mann *et al* compile results of the modelling across 21 countries and discuss patterns and insights derived from the studies.^15^ Finally, Spencer *et al* explore the equity implications of potential tobacco tax increases in 19 countries, demonstrating how tobacco control is linked to addressing poverty and inequalities.^16^ Taken together, the papers provide a view of the FCTC 2030 project’s contribution to examining and strengthening responses to the health, social and economic aspects of tobacco.

### Objectives of the WHO FCTC and FCTC 2030 project

The WHO FCTC (2005) is an evidence-based UN treaty aimed at supporting countries to reduce tobacco use. There are 183 Parties to the WHO FCTC, covering over 90% of the world’s population and fostering solidarity for tobacco control. The WHO FCTC includes a range of treaty obligations for Parties to implement, covering a wide range of tobacco control measures including reduction of demand for tobacco, reduction of the supply of tobacco, protection of the environment, liability, scientific and technical cooperation, and communication of information. Implementation of WHO FCTC measures often requires engagement of actors outside health, for example, ministries of finance are needed to design and implement effective tobacco taxes and ministries of education have a role in raising health literacy among young people on tobacco use. The WHO FCTC, therefore, includes obligations to strengthen governance across sectors, including in the areas of planning, coordination, financing and protection against tobacco industry interference in policy-making.

While nearly all WHO FCTC Parties have implemented at least some of the Treaty’s measures, most have not achieved full implementation.^17^ This owes to a range of technical, financial and political barriers that include limited or lack of effective cooperation across crucial actors and sectors; concerns related to the cost of implementation; potential economic losses from reduced tobacco production and sales; real or perceived non-acceptance of tobacco control by public and private stakeholders; and interference by the tobacco industry and its affiliates, for example, in the policy areas of taxation, trade and investment, and employment from farming and manufacturing tobacco.^5 18^ Reinforcing these barriers is low awareness of the full, society-wide harms of tobacco, particularly in LMICs facing or at risk of high burdens.^19^

The FCTC 2030 project is coordinated by the Secretariat of the WHO FCTC and delivered together with United Nations Development Programme and WHO. Countries have been selected through an open application process to receive catalytic technical and financial assistance, in line with the Global Strategy to Accelerate Tobacco Control: Advancing sustainable development through implementation of the WHO FCTC 2019–2025.^20^ The selection criteria for inclusion in the project included a Party’s ambition and readiness to accelerate implementation of the WHO FCTC.^21^

In their project applications, FCTC 2030 countries requested a varied range of support within the priority areas of the project including the treaty’s time-bound measures, taxation and governance obligations. Every project country, regardless of its progress on tobacco control, requested a tobacco control investment case. This reflects the need and desire to identify and finance the most cost-effective solutions and to combat economic misinformation around tobacco control.

## Method: the tobacco control investment cases

**Figure 1:**
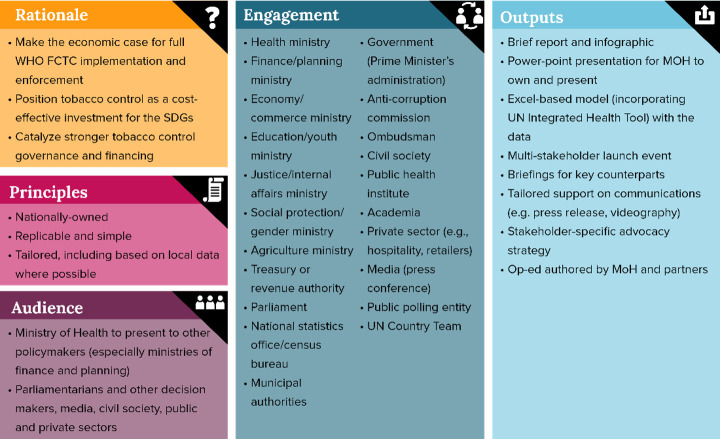
Key aspects of the tobacco control investment cases. FCTC, Framework Convention on Tobacco Control; MOH, Ministry of Health.

The cases empower governments and their partners with data and evidence on the national costs of tobacco use, the benefits of tobacco control and politically viable pathways for stronger WHO FCTC implementation. They have two components: an economic analysis and an institutional and context analysis (ICA). The economic component assesses: (1) the economic burden of tobacco use by analysing the costs of tobacco use to society, the health sector and economy, (2) the costs for the country to strengthen implementation and/or enforcement of key WHO FCTC demand-reduction tobacco control policy actions, (3) the health and economic benefits of taking these measures and (4) the return-on-investment of the interventions over a 5-year and 15-year period. The ICA employs desk-based research and key informant interviews with in-country stakeholders to discern socio-political barriers and opportunities for the economic findings to be understood, accepted and acted on. The process promotes whole-of-society engagement from the start. For example, ministries of finance and labour, not just ministries of health, partake in data collection. To encourage wide dissemination of findings, the investment cases are ‘handed over’ to governments at high-level national events that typically involve non-health sectors, civil society, academia and media. As the investment cases and associated outputs (eg, infographics) are published, they enable ministries of health and other stakeholders to advocate within the country, including to parliamentarians. Figure 1 summarises key aspects of the investment cases.

The cases have multiple objectives and uses around the overarching goal of strengthening implementation of the WHO FCTC (box 1).

Box 1 **Objectives and uses of tobacco control investment cases**

- Provide decision-makers with access to better data and evidence for action.
- Support the passing, enforcement and oversight of effective laws, policies and regulatory measures.
- Justify increased budget allocations for tobacco control and direct financing toward the most cost-effective options.
- Position stronger tobacco taxes as a sustainable, government-led financing option.
- Elevate tobacco control as a national priority, demonstrating its relevance to achieving the Sustainable Development Goals and supporting its inclusion in sectoral and national plans and financing frameworks.
- Inform the development, costing, implementation and enforcement of ambitious and prioritised national multisectoral policies and plans for tobacco control.
- Support the establishment or strengthening of effective national and/or subnational coordination mechanisms, including by focusing multisectoral efforts on high-impact interventions.
- Combat tobacco industry interference in policy-making by sensitising key ministries, improving multisectoral planning and coordination, and recommending good practices to protect public health policy-making from undue influence.
- Raise public and political awareness of the benefits of WHO Framework Convention on Tobacco Control (FCTC) implementation, and strengthen engagement and ownership of leaders, non-health ministries, parliamentary unions/associations and civil society, including in public discussions and decision-making processes.
- Strengthen international and multilateral cooperation to support treaty implementation, including through coordinated support with the Secretariat of the WHO FCTC and interagency coordination at global, regional and country levels.

## Results: observed tobacco control advancements in the 21 countries

The FCTC 2030 project countries with completed tobacco control investment cases have recently advanced WHO FCTC implementation. To date, the authors have identified 30 actions that align with investment case findings and recommendations across 17 of the 21 countries (table 1).

**Table 1:** Recent tobacco control advancements in the investment case countries

	Tobacco taxes	Smoke-free policies	Public awareness	TAPS ban	Warning labels	Plain packaging	Cessation services	Strategy, coordination and engagement
Armenia (2021)		Restrictions banning smoking in nearly all enclosed public places (2022)		Introduced a comprehensive ban on tobacco advertisement in 2022				
Cabo Verde (2019)	Increased excise taxes on tobacco (2020)						Smoking cessation programme launched and scaled (2020–2021)	
Cambodia (2019)								
Chad (2021)								Issued presidential decree on preventing tobacco industry interference (2019)
Colombia (2021)			New pictorial health warnings (2020)					Multistakeholder dialogue on tobacco taxes (2020)
Costa Rica (2023)	Tax on vaping devices and accessories (2021)	Banned vaping and e-cigarettes in most public places (2021)						
El Salvador (2020)								
Eswatini (2021)	Increased customs tax on imported tobacco products (2019)							
Georgia (2019)		Banned smoking in enclosed work and public places (2017)		New restrictions on advertisement and display of tobacco products (2017)		Plain packaging of tobacco products (2017)		Protections from tobacco industry interference in policymaking (2017)
Ghana (2023)	Increased excise tax on tobacco products (2022)							
Jordan (2021)			National anti-tobacco campaign (2019)				Initiative for access to smoking cessation services (2020)	Established national committee to support FCTC 2030 (2020)
Laos (2022)								Multistakeholder discussions on tobacco tax increases (2021)
Madagascar (2019)								
Myanmar (2018)						Plain packaging of tobacco products (2022)		Discussions on tobacco tax increases (2018)
Nepal (2019)								Discussions on tobacco tax increases (2020)
Samoa (2021)		Restrictions on sale and use of electronic nicotine and non-nicotine delivery systems (2019)						Established National Tobacco Control Committee (2019)
Sierra Leone (2021)		Restrictions on smoking in enclosed public spaces (2022)		Comprehensive ban on advertisement of tobacco and nicotine products (2022)				New mechanisms for coordination and planning
Sri Lanka (2019)								Announced plans to introduce plain packaging requirements (2019)
Suriname (2021)								
Tunisia (2021)				Mandated that graphic warning labels cover 70% of the outer packaging of tobacco products (2022)				
Zambia (2021)								New mechanisms for coordination and planning

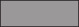
Not modelled due to data or methodology limitations; 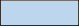 Not modelled as advanced implementation already achieved; 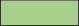 Implemented measures observed following the investment case.

The non-shaded cells refer to no measures observed following the investment case.

The year following each country name in the left column is the date the investment case was completed.

FCTC, Framework Convention on Tobacco Control; TAPS, tobacco advertising, promotion, and sponsorship.

Some of these actions occurred after initiation of the investment case process and before its completion. The advancements include stronger implementation of the demand-reduction measures. Specifically, four countries strengthened their tobacco tax regime; five countries instituted stronger smoke-free policies; two countries made changes to raise awareness of the harms of tobacco use; four countries instituted greater limits on tobacco advertising, promotion and sponsorship; one country legislated for plain packaging for tobacco and two countries established tobacco cessation programmes.

The observed tobacco control advancements also include strategy, coordination and multistakeholder engagement in line with WHO FCTC Article 5. Eight countries formed institutions and activities to coordinate and strengthen tobacco control planning and engage stakeholders. Two countries took legislative action to prevent tobacco industry interference in policymaking. Such core governance improvements not only make tobacco control policy progress more likely but also more likely to be sustained and resourced into the future. That includes the importance of evidence-based multisectoral planning and coordination to tackling tobacco industry interference in policy-making.^22^

Many countries deployed combinations of policies to achieve greater effectiveness. An example is Georgia where the Ministry of Health (MoH) used its investment case to support the adoption and partial implementation of comprehensive tobacco control legislation. The MoH presented findings at parliamentary hearings relating to the implementation of the WHO FCTC.^23^ By prohibiting smoking in enclosed work and public places, comprehensively banning tobacco advertising, promotion, and sponsorship and introducing plain packaging requirements, Georgia’s landmark tobacco control law makes the country a leader in protecting people from the harms of tobacco.^24^ At the same time, Georgia also illustrates the need for sustained tobacco control efforts: the standardised packaging regulations are yet to take effect (as of 2024) following a series of delays and proposed changes that would limit their public health impact.^25^

## Discussion

A careful review of the results and experiences of the authors in conducting the 21 investment cases, including feedback from in-country advocates and government officials, provides clear learnings and reflections from the multiyear effort.

The investment cases appear to contribute to a diverse range of impacts and uses. FCTC 2030 countries have shown multiple tobacco control advancements that are plausibly attributable in part to the investment cases. This finding affirms the demand for and value in revealing both the society-wide harms of tobacco use and the economic benefits of stronger control. It reinforces the mixed-methods evaluation of FCTC 2030 that found the cases to be ‘a valuable input’ which ‘built understanding of the economic impact of tobacco use and the benefits of its control’, offered ‘clearly new (perspectives) to many government officers’ and, for example, ‘helped in prioritising tobacco control within the Ministry of Finance in Jordan and supported the case for increased taxation in Colombia’.^26^ Another review of four countries in the Americas also found that the FCTC 2030 investment cases can help to strengthen tobacco control, by increasing public and political support for implementation of the WHO FCTC and by informing effective planning, legislation, coordination and financing.^27^

There is still more to learn about how the investment cases are being and can be used, to what effect and for how long (ie, ‘shelf life’). Because multiple variables influence tobacco control, there is a need to more deeply examine the contributions of the investment cases to the observed policy advancements in each country. This is particularly true in contexts with long histories and wider ecosystems of tobacco control work. Recognising that tobacco control policy progress is often non-linear, it is also crucial to better understand how the cases can contribute to not just policy advancements but also to the strengthening of WHO FCTC Article 5 as a bedrock for sustained efforts. That must include a forward-looking, not just retrospective, analysis. For example, continued and more systematic engagement from the UN and partners following an investment case handover is crucial to comprehensively assess barriers, opportunities and additional technical support needs around achieving fuller implementation of investment case recommendations. To sustain country ownership, it is also important to build national capacities and encourage national experts to re-run the analysis over time and in the context of changing national circumstance such as government turnover.

It may be critical to embed investment cases within broader support for WHO FCTC implementation. The investment cases were requested by FCTC 2030 project applicants that needed to convey readiness to accelerate WHO FCTC implementation and received additional project assistance. These factors may have played a role in the observed tobacco control advancements. Indeed, additional FCTC 2030 project assistance covers areas that are mutually reinforcing with investment analysis such as policy development and implementation (including compliance building and enforcement), fiscal and legislative processes; costed and prioritised strategic planning; domestic resource mobilisation; and multisectoral planning and coordination. Moving forward, greater complementary support is needed to address tobacco industry interference in policy-making, including to counter biased information from the industry. The authors observed just two tobacco control advancements specific to this area, despite the investment cases typically including recommendations on WHO FCTC Article 5.3 (eg, codes of conduct for government officials). Moreover, tobacco taxation consistently emerged in the cases as having among the highest returns on investment (ROI) and has advanced in at least four of the 21 countries. While this is notable progress given underutilisation of stronger tobacco taxes globally, that more of the countries have not advanced stronger tobacco taxes despite the evidence provided is also a sign of powerful tobacco industry influence.^28^

The appeal and perceived effectiveness of the cases likely owes to their core principles. Though tobacco control is evidence-based and tried, tested, and reliably effective across contexts, the FCTC 2030 project countries have demonstrated demand for tailored, comprehensive, inclusive and country-owned and led investment cases. Despite common barriers to tobacco control, policy-makers in countries may draw limited evidence from global analyses or the experiences of other countries. The investment cases recognise that while all WHO FCTC Parties are oriented around the implementation of a global treaty, they are at different junctures of their tobacco control journey. Parties all have different burdens, capacities, contexts and histories. The cases provide governments flexibility in selecting which interventions to analyse, at what intensity and with what data. That includes space for countries to select custom, nationally relevant analysis (eg, alternative livelihoods for tobacco farmers in Zambia). The investment cases also seek to identify and complement the unique attributes of each context, including other ongoing tobacco control efforts. In Colombia, for instance, that included engaging local researchers and leveraging a strong public health communications apparatus to highlight the dangers of tobacco smoke exposure for children.^27^

The ICA is crucial for giving the cases further depth, authority and resonance. While the economic analysis tends to receive the headlines in communications, the ICA works behind the scenes through stakeholder and narrative analysis to increase the chances of the investment case findings and recommendations being understood, accepted and acted on. As part of the national tobacco control investment cases, tailoring messaging, giving stronger voice to champions, and countering powerful opponents. They have positioned tobacco control as an accelerator, not competitor, of other national priorities and interests. They have also suggested near-term policy windows for action together with opportunities in planning, coordination and financing across sectors. In Georgia, for instance, investment case findings and recommendations were contextualised within tobacco control lawmaking efforts. Other examples include connecting tobacco taxation with broader economic policy in Ghana, and linking tobacco control with the protection of women, youth, the poor, and children in Samoa.

The investment case approach is iterative; it can and must be continually strengthened. From the very first case, FCTC 2030 project partners have welcomed and attempted to incorporate feedback on the approach while maintaining credibility and replicability. Nugent *et al* 14 detail methodological enhancements over time. Examples of other steps taken or envisioned to strengthen the investment case approach include supporting national authorities to assess how cigarette tax revenue may change with alterations to cigarette tax structure or rate; incorporating analysis on how tobacco control impacts illicit trade and livelihoods, to interrogate additional economic considerations; and providing support in integrating investment *cases* into investment *frameworks* which directly connect the economic findings with costed planning, budget analysis and financing for health and the SDGs. There is also impetus for the cases to delve deeper on gender, equity and rights in the context of economies of well-being.

The tobacco control investment cases have been an exemplar for other NCD risk factors to build a case for prevention and control. Since the initiation of the tobacco control investment cases and other UN support on NCD investment cases, appetite for economic data among health authorities has continued to grow.^29^ Countries have requested and are receiving investment case support for other issues including mental health, pollution, alcohol, nutrition and road safety. As with the tobacco control cases, these other issue-specific cases are critical for encouraging investment of dedicated resources, prioritising the most cost-effective solutions, and strengthening prevention and control. However, they may also entrench siloed approaches at a time when evidence of interactions between health conditions and their risk factors is increasing.^30 31^ To optimise planning and financing in the face of multiple health priorities and the need to advance universal health coverage, decision-makers and budget holders would benefit from more integrated analysis that examines how ROIs across issue-specific cases may relate and interact. It is also important to ensure that the cases communicate with other economic analysis that is relevant in a given context.^32^ One limitation in doing so relates to available data. For tobacco control investment cases, the evidence base and sources of data that contribute to the analyses are more readily available than for some other investment cases.

### Limitations

This paper faces several limitations. It is challenging to draw conclusions about policy change attribution, and we have not sought here to analyse the causal chain affecting tobacco policy in 21 countries. Rather, the paper collates and assesses near-term tobacco control actions during and following investment case development. Countries that started and completed investment cases earlier have had more time for the cases to affect policy decisions than countries that followed later. The paper conveys that—taken together—recent policy changes and other tobacco control advances that occurred in many of the 21 investment case countries may plausibly be related to the investment case analysis. We claim that the investment cases likely contributed to the observed tobacco control advancements. We believe this to be a conservative claim given the demand for the investment cases, that the observed actions align with case findings and recommendations, and corroborative evidence of impact through an independent evaluation of the FCTC 2030 project.^26^

## Conclusion

This collection of papers describing the national tobacco control investment cases as part of the FCTC 2030 project establishes a strong evidence base for advancing national-level tobacco control. Taken together, these papers offer readers a deep understanding of the potential for intensified tobacco control to produce positive economic benefits in the countries that were studied and, by implication, other countries wishing to reduce the malign effects of tobacco use.

## Data Availability

Data sharing not applicable as no datasets generated and/or analysed for this study.
